# The transcriptomic response of bovine uterine tissue is altered in response to sperm from high- and low-fertility bulls^†^

**DOI:** 10.1093/biolre/ioad031

**Published:** 2023-03-22

**Authors:** Eimear M Donnellan, Paul Cormican, Colm Reid, Gina Duggan, Miriama Stiavnicka, Kieran G Meade, Sean Fair

**Affiliations:** Laboratory of Animal Reproduction, Department of Biological Sciences, Biomaterials Research Cluster, Bernal Institute, Faculty of Science and Engineering, University of Limerick, Limerick, Ireland; Animal & Bioscience Research Department, Teagasc Animal & Grassland Research and Innovation Centre, Grange, Dunsany, Co., Meath, Ireland; UCD Veterinary Sciences Centre, Dublin, Ireland; UCD Veterinary Sciences Centre, Dublin, Ireland; Laboratory of Animal Reproduction, Department of Biological Sciences, Biomaterials Research Cluster, Bernal Institute, Faculty of Science and Engineering, University of Limerick, Limerick, Ireland; School of Agriculture and Food Science, University College Dublin, Dublin, Ireland; Laboratory of Animal Reproduction, Department of Biological Sciences, Biomaterials Research Cluster, Bernal Institute, Faculty of Science and Engineering, University of Limerick, Limerick, Ireland

**Keywords:** spermatozoa, immune response, endometrium, bovine, interleukin-1 alpha

## Abstract

Despite stringent quality control checks, some bulls with apparently normal semen quality yield lower than expected pregnancy rates. This study profiled the transcriptome and performed histological analysis of the bovine uterus in response to sperm from high-fertility (HF) and low-fertility (LF) bulls. Postmortem uterine biopsies and uterine explants were collected from heifers 12 h after a fixed time artificial insemination (AI) to a synchronized estrus with frozen–thawed semen from five HF (fertility rate 4.01% ± 0.25) and five LF (fertility rate − 11.29% ± 1.11; mean ± SEM) bulls. Uterine biopsies were also collected from control (CTRL) heifers, which were not inseminated. RNA-sequencing and histological analysis were performed for differential gene expression and neutrophil quantification. In the HF treatment relative to CTRL heifers, there were 376 genes significantly differentially expressed in the endometrium with just one gene differentially expressed in the LF treatment relative to CTRL heifers. Comparing the HF and LF treatments directly, there were 40 significantly differentially expressed genes (*P* < 0.05). Transcriptomic analysis shows a predominant role for the inflammatory marker Interleukin-1 alpha, which was further confirmed by immunohistochemistry. Quantification of neutrophils in the endometrium showed a significant effect of sperm; however, there was no difference in neutrophil numbers between HF and LF groups. In conclusion, this novel study clearly shows a distinct inflammatory response to sperm in the endometrium and a divergent transcriptomic response to semen from HF and LF bulls.

## Teaser

The concept that sperm from high fertility bulls are priming the endometrium for implantation and a subsequent pregnancy merits further investigation.

## Graphical Abstract

**Graphical Abstract:**
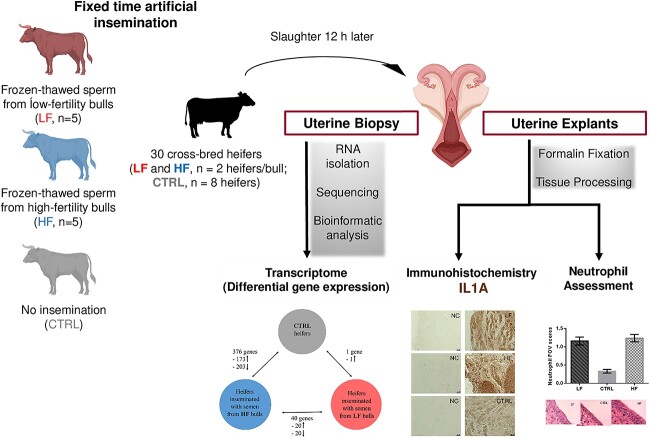

## Introduction

Artificial insemination (AI) is used to improve the genetic merit of herds as one bull with desirable traits can be used to inseminate thousands of cows. Prior to the release of semen into the field animal breeding centers worldwide, a number of microscopy-based prefreeze and post-thaw quality control checks were typically performed [1]. Despite these stringent controls, some bulls with apparently normal semen quality yield unacceptable low pregnancy rates, which can have significant economic losses for farmers. The reason for this is largely unknown, and most studies have focused on attempting to predict bull fertility using a range of *in vitro* sperm functional parameters combined with various statistical models [2, 3]. These *in vitro* assays range from microscopic evaluations to flow cytometry analysis of plasma membrane integrity, oxidation level, mitochondrial status, and sperm chromatin structure, to name but a few [4–6]. These studies, however, do not attempt to assess where along the developmental pathway reproductive wastage occurs for some bulls.

During AI, semen is deposited in the uterus where sperm interact with the uterine and oviductal epithelia as well as the mucus secretions before reaching the site of fertilization in the ampulla [7]. In many species to-date, it has been shown that semen (sperm and seminal plasma) is capable of initiating an immune response in the uterus and has been best described in mice, pigs, horses, and more recently in cattle [8–10]. Bovine *in vitro* studies have shown sperm alone can induce a pro-inflammatory response in the uterus [11] mediated by the TLR2/4 pathways [12, 13]. *In vivo* sperm that have come in contact with seminal plasma (SP), but not SP alone, induce transcriptional changes in the uterus after natural mating in cattle [10]. Studies in mice have shown that the interaction of sperm with the uterine epithelial cells help initiate the female immune response in early pregnancy and is mediated by*IL6, CXCL2,* and *CSF3* [14]. Infertility in mice has been linked with a lack of *IL11A* and a defective uterine response to implantation [15]. Genes expressed in the bovine endometrium associated with high-fertility (HF) compared to low-fertility (LF) heifers include *DKK1, IGFBP1, and MEP1B* [16]; however, the effect of sire fertility on the endometrial expression and pregnancy success rate remain to be elucidated. An *ex vivo* model showed bull sperm enter the uterine glands where they interact with polymorphonuclear leukocytes (PMNs) [17]. Postinsemination, PMNs are known to rapidly enter the uterine lumen of cows. The route of this PMN migration to the uterus is via uterine glands where they phagocytize sperm or form neutrophil extracellular traps [18]. The role of this immune response appears to clear the endometrium of excess sperm and bacteria and to prepare the endometrium for pregnancy [8]. The level of inflammation and PMN migration, which is beneficial to priming the uterus for the subsequent pregnancy, is a complex process, and some studies suggest that moderate influx of PMNs leads to significantly higher conception rates [19, 20].

We hypothesized that sperm from bulls of differing fertility phenotypes elicit a differential immune response, which can affect outcomes by altering sperm transport and/or by differential priming of the uterus, which may affect embryo development once the blastocyst comes back down into the uterus approximately five days of postfertilization. To address this, we used a molecular-based approach to investigate the uterine biology of uterine interactions *in vivo* with sperm from HF and LF AI bulls.

## Materials and methods

### Ethical approval

Protocols were developed in accordance with the Cruelty to Animals Act (Ireland 1876, as amended by European Communities regulations 2002 and 2005) and the European Community Directive 86/609/EC. All animal procedures were conducted under experimental license from the Health Products Regulatory Authority and approved by the University of Limerick Animal Ethics Committee.

### Experimental model

The estrus cycles of thirty, cross-bred heifers were synchronized and inseminated using a fixed-time AI protocol. An eight-day intravaginal progesterone device (PRID Delta) was used with gonadotrophin-releasing hormone (Ovarelin; 2 ml) administered intramuscularly at the time of PRID insertion. All heifers were administered with prostaglandin F2 alpha (Enzaprost; 5 ml) intramuscularly to induce luteolysis 24 h prior to PRID removal. Two heifers lost the PRIDs and were removed from the experiment. Heifers received a single fixed time insemination of frozen–thawed semen at 72 h post-PRID removal or were not inseminated and left as part of a control group. Ovarelin (2 ml) was administered intramuscularly to all heifers at the time of AI.

Heifers were allocated to one of three treatments and were inseminated by a single trained technician with semen from five HF bulls (2 heifers/bull) and five LF bulls (2 heifers/bull) or not inseminated bulls (CTRL; *n* = 8 heifers). Two semen straws (one from each of two ejaculates) from each bull were used. The study was carried out over two replicates.

### Bull selection

Data on the field fertility of a population of Holstein-Friesian bulls (*n* = 840) used in Ireland were obtained from the Irish Cattle Breeding Federation (ICBF) database. Each bull had a minimum of 500 inseminations based on an adjusted sire fertility model [21]. Adjusted bull fertility was defined as pregnancy to a given service identified retrospectively either from a calving event or where a repeat service (or a pregnancy scan) deemed the animal not to be pregnant to the said service. Cows and heifers that were culled or died on farm were omitted. These raw data were then adjusted for factors including semen type (frozen, fresh), cow parity, days in milk, month of service, day of the week when serviced, service number, cow genotype, herd, AI technician, and bull breed and were weighted for number of service records resulting in an adjusted pregnancy rate centered at 0%. For this study, bulls classified as having HF had an average adjusted fertility score of +4.01 ± 0.25% (*n* = 5; average no. inseminations per bull = 38 041: Table 1), whereas, those classified as LF had an average of −11.29 ± 1.11% (*n* = 5; average no. inseminations = 978; Table 1).

**Table 1:** *In vivo* fertility data of Holstein Friesian bulls of HF and LF as determined by an adjusted animal model (AAM). Mean of the population in the AAM was 0%.

**Bull**	**Number of inseminations**	**AAM (%)**	**Fertility status**
1	1479	−15.53	Low
2	519	−12.13	Low
3	609	−10.33	Low
4	1772	−9.83	Low
5	510	−8.93	Low
6	12 417	+3.57	High
7	5121	+3.67	High
8	37 856	+3.87	High
9	99 953	+3.97	High
10	34 859	+4.97	High

### Tissue collection

Twelve hours after insemination, heifers were slaughtered at a commercial abattoir. Reproductive tracts were collected immediately postmortem, and the uterine horn ipsilateral to the ovulated ovary was opened longitudinally with a sterile scissors. Tissue samples were obtained from the intercaruncular area of the uterine horn close to the side of the uterine body using a sterile 8-mm biopsy punch (Stiefel Laboratories Ltd, High Wycome, UK). Sterile blades were used to dissect the endometrium away from the myometrium. Samples were immediately stored in liquid nitrogen, transported to the laboratory, and stored at −80°C.

### RNA extraction, library prep, and RNA sequencing

One uterine biopsy from each heifer was selected for RNA transcriptomic analysis. Samples were homogenized in Trizol, followed by RNA extraction using an RNeasy Mini Kit (Qiagen, Hilden, Germany) as per manufacturer’s instructions. The quantity of RNA was determined using the Nano Drop 1000 (Thermo Fisher Scientific, WA, USA) and quality with the Agilent Bioanalyzer (Agilent Technologies, CA, USA). The average RNA integrity number value of all samples was >7. TruSeq (Illumina TruSeq stranded mRNA library construction) RNA libraries were prepared for all 28 samples. All libraries were sequenced over Illumina NovaSeq sequencer, generating 100 bp paired end reads (50 million reads/sample).

### Quality control, mapping, and differential read count quantification

Raw sequence reads were downloaded in FASTQ format, and these sequence reads were quality assessed using software fastqc (v.0.11.8) (http://www.bioinformatics.babraham.ac.uk/projects/fastqc/). Sequences from all samples were quality trimmed and cleaned of adaptor sequences using BBDuk java package. On average, 0.2% of the bases were trimmed per sample. Trimmed reads were mapped to the Bovine Reference Genome in ARS-UCD1.2 using STAR RNA-seq aligner v2.5.2, and uniquely mapped read counts per Ensemble annotated gene/transcript were estimated using the STAR–quant Mode option. Genes with zero read counts across all samples as well as nonprotein coding genes were removed prior to subsequent analysis.

Differential gene expression analysis and data transformations and visualization were carried out using DeSeq2 v1.30 in R 4.0.2. Sample clustering was carried out on variance stabilizing transformed data and visualized using PCA. Differentially expressed gene (DEG) lists were generated using a negative binomial generalized linear model and pairwise comparisons using each combination of the uterine biopsy groups. *P* values were adjusted for multiple comparisons using a Benjamini and Hochberg (B–H) method. Genes with an adjusted *P* value <0.05 were considered differentially expressed and used for further data exploration and pathway analysis. Gene Ontology (GO) and KEGG pathway analysis of DEGs were carried out using cluster Profiler (v3.18.0) [22].

### Neutrophil assessments and quantification

The ability of neutrophils to move from the mucosal region of the endometrium through the epithelium in response to the external environment was assessed as follows:

Endometrial samples (~1 cm^2^), which included the surface epithelium, were harvested from three animals for each group (heifers exposed to LF semen, heifer exposed to HF, and CTRL heifers—no semen exposure) within 30 min of slaughter and fixed in 10% neutral buffered formalin for 48 h. Following processing into paraffin, tissue blocks were sectioned at 5 μm thickness using a Leica microtome and stained with hematoxylin and eosin. The sections were examined using an Olympus BX43 microscope fitted with an image analyser (Image—Pro Premium; Media Cybernetics, MD, USA). Using a 63x oil immersion objective, 20 and 40 fields of view (FOV) were selected per sample. Neutrophils within the columnar epithelium region only were quantified. The count was expressed as the average number of neutrophils per FOV per group. On average, 106 FOV were analyzed per treatment group.

### Endometrial expression of IL-1 alpha

Uterine biopsies from three heifers from each treatment were used for immunohistochemical localization IL-1 alpha. The formalin-fixed paraffin-embedded endometrial biopsies were sectioned at 5 μm thickness and stained using primary antibody Interleukin-1 alpha (#P420A, Thermo Fisher Scientific). Samples underwent antigen retrieval in 0.01 M citrate buffer and were blocked for endogenous peroxidases using 0.3% H_2_O_2_. A horseradish-peroxidase-conjugated goat anti-rabbit IgG H&L secondary antibody (Abcam, Cambridge, UK) was applied and developed with 3,3’diaminobenzidine substrate (DAB substrate, Abcam) before being mounted. Representative pictures were taken using an Olympus BX43 microscope fitted with an image analyser (Image—Pro Premium; Media Cybernetics). A negative control with no primary antibody was applied.

## Results

### Comparison 1: significant differential expression of genes in the endometrium of heifers in response to semen from HF bulls relative to control heifers

**Figure 1:**
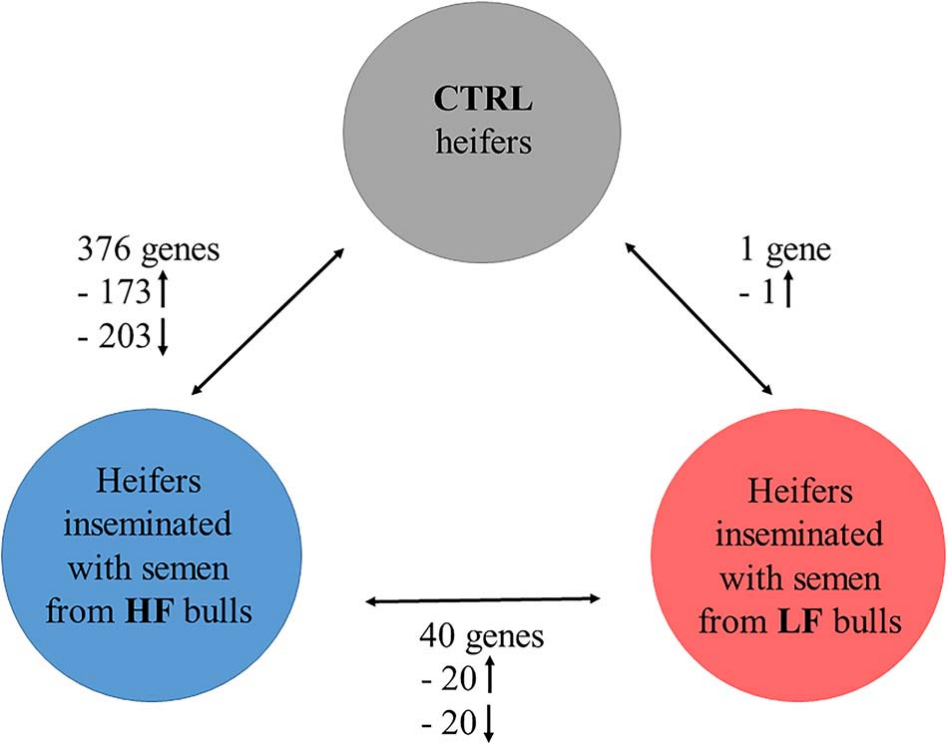
Number of DEGs in the endometrium of heifers where three comparisons were performed. (i) Heifers inseminated with frozen–thawed semen from HF bulls (*n* = 10 heifers; 2 heifers/bull) relative to the CTRL (*n* = 8 heifers). (ii) Heifers inseminated with semen from LF bulls (*n* = 10 heifers; 2 heifers/bull) relative to CTRL. (iii) Heifers inseminated with HF bulls relative to LF bulls. Gene expression was determined by RNA-seq using an adjusted *P* < 0.05 and FC > 1. Arrows represent the direction of fold change.

**Figure 2:**
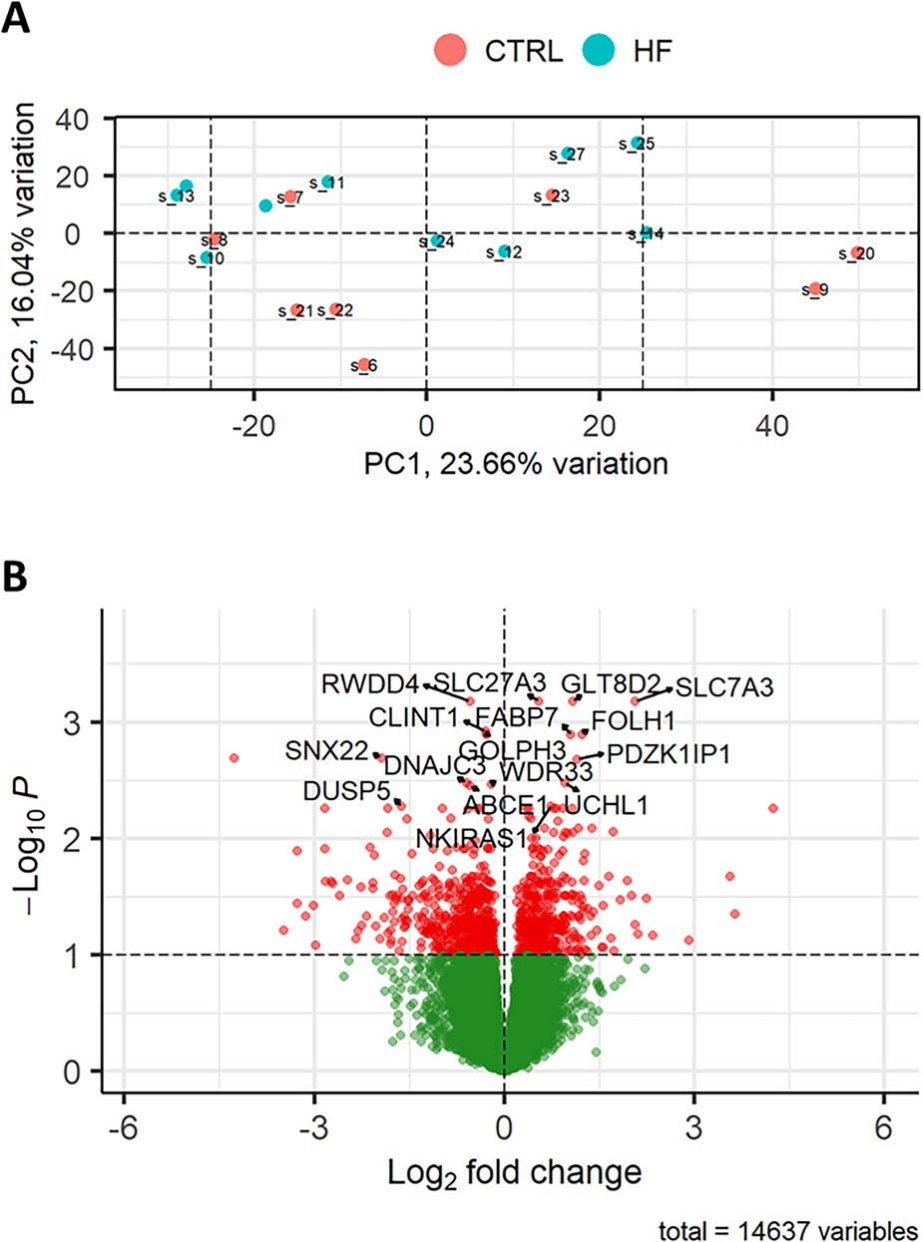
DEGs in the endometrium of heifers inseminated with frozen–thawed semen from HF bulls (*n* = 10 heifers) relative to the CTRL noninseminated heifers (*n* = 8 heifers). (A) PCA plot showing the distribution of RNA-seq samples, where colors indicate the two treatments and numbers refer to specific heifer IDs (as detailed in Supplementary file 1). (B) Gene expression data are presented as a volcano plot using log values of the fold change and *P* value. Each data point represents a single gene, with those in red representing genes that survived the cut off thresholds of adjusted *P* < 0.05. The most significant DEGs are labelled, where gene names are available.

Using rigorous statistical filtering, 376 genes were identified as significantly differentially expressed (adjusted *P* value <0.05) in the endometrium of heifers inseminated with semen from HF bulls relative to the CTRL heifers (Figure 1). There was clear separation between samples in both groups as shown in the PCA plot (Figure 2A). Of the 376 DEG’s, there were 173 genes with increased expression in the HF treatment and 203 with decreased expression in the HF treatment group, and this relatively equal of divide was also evident from the volcano plot (Figure 2B). The fold change values, which were increased in HF compared to CTRL treatments, ranged from 1.15- to 21.99-fold. The full list of DEG is available in Supplementary file 2, and the top ten most significant DEGs are shown in Table 2. The gene with the largest fold change 21.99 was *GABRA4,* which is involved in transmembrane signaling receptor activity*.* The top genes with increased expression in HF treatments included *PTGDS, GABRA4*, and *SLC7A3.* Of the 173 upregulated genes, there were multiple genes encoding coiled-coil domain containing proteins (*CCDC114, CCDC28B, CCDC62, CCDC80,*) with genes from this family involved in protein folding [23]. In relation to immune genes, there was increased expression of *IL11RA, IL4I1*, and *IL1A.*

**Table 2:** Top 10 DEGs in the endometrium of heifers inseminated with frozen–thawed semen from HF bulls relative to CTRL heifers. Positive absFC values indicate higher expression in HF relative to CTRL, and negative values indicate higher expression in CTRL relative to HF.

**Gene symbol**	**Ensembl ID**	**Gene Name**	**Gene functions**	**absFC**
ALOX12	ENSBTAG00000021933	Arachidonate 12-lipoxygenase	Lipoxygenase activity	−5.90
PTGDS	ENSBTAG00000015074	Prostaglandin D2 synthase	Prostaglandin D synthase activity	2.15
GABRA4	ENSBTAG00000016645	Gamma-aminobutyric acid type A receptor alpha4 subunit	Transmembrane signaling receptor activity	21.99
GLT8D2	ENSBTAG00000000925	Glycosyltransferase 8 domain containing 2	Transferase activity	2.11
RWDD4	ENSBTAG00000003081	RWD domain containing 4	Protein binding	−1.46
SLC7A3	ENSBTAG00000007403	Solute carrier family 7 member 3	Transmembrane transporter activity	4.19
SLC27A3	ENSBTAG00000021862	Solute carrier family 27 member 3	Catalytic activity	1.46
CLINT1	ENSBTAG00000016199	Clathrin Interactor 1	Transport between the trans-Golgi network and endosomes	−1.23
FOLH1	ENSBTAG00000007397	Folate hydrolase 1	Folate hydrolase and N-acetylated-alpha-linked-acidic dipeptidase (NAALADase) activity	2.33
GOLPH3	ENSBTAG00000032848	Golgi phosphoprotein 3	Regulatory role in Golgi trafficking	−1.22

Of the 203 significantly downregulated genes in the endometrium of HF heifers relative to the CTRL treatment, the fold change ranged from −19.23 to −1.12. The most significantly decreased gene in the HF group was *ALOX12*, which is known to regulate platelet aggregation and cell migration as well as inflammation and apoptosis [24]. Multiple genes encoding solute carrier (SLC) proteins (*SLC13A5, SLC22A3, SLC39A14, SLCO2A1, SLCO4A1*) were decreased in the HF treatment heifers.

The top GO molecular function was peptide binding, signaling receptor activator activity, and receptor ligand activity (Table 3).

**Table 3:** GO and molecular function for heifers inseminated with frozen–thawed semen from HF bulls relative to control heifers.

**ID**	**Description**	**Gene ratio**	**Bg ratio**	***P* value**	**p.adjust**	**geneID**	**Count**
GO:0042277	Peptide binding	18/322	202/12367	0.0000	0.0031	ANPEP/TRHDE/ CEMIP/ APOA1/ EDNRB/IDE/ VIPR2/ DHCR24/KPNB1/ PTGES/ DLGAP3/ FOLH1/LDLR/ EBI3/ITM2C/ OXTR/IPO4/SSTR1	18
GO:0030546	Signaling receptor activator activity	18/322	259/12367	0.0002	0.0285	FGF18/HMGB2/IL6/ APOA1/ CCL2/GDF10/ TNFSF18/ IL1A/WNT16/ STC1/CSF1/ PTN/IGF1/ CDK5/PTHLH/ CCL25/ INHA/EBI3	18
GO:0048018	Receptor ligand activity	17/322	252/12367	0.0003	0.0461	FGF18/HMGB2/IL6/APOA1/ CCL2/GDF10/TNFSF18/ IL1A/WNT16/STC1/CSF1/ PTN/IGF1/PTHLH/CCL25/ INHA/EBI3	17

**Gene ID** = list of genes assigned to a given pathway.

**Bg ratio** = the ratio of the number of genes that are not differentially expressed in a pathway to the number of expressed genes with GO identifier.

**Gene ratio** = the ratio of the number of differentially expressed genes in a given pathway to the number of differentiating genes with GO identifier.

**p. adjust** = adjusted *P* value.

### Comparison 2: minor but significant differential expression of genes in the endometrium of heifers in response to semen from LF bulls relative to control heifers

**Figure 3:**
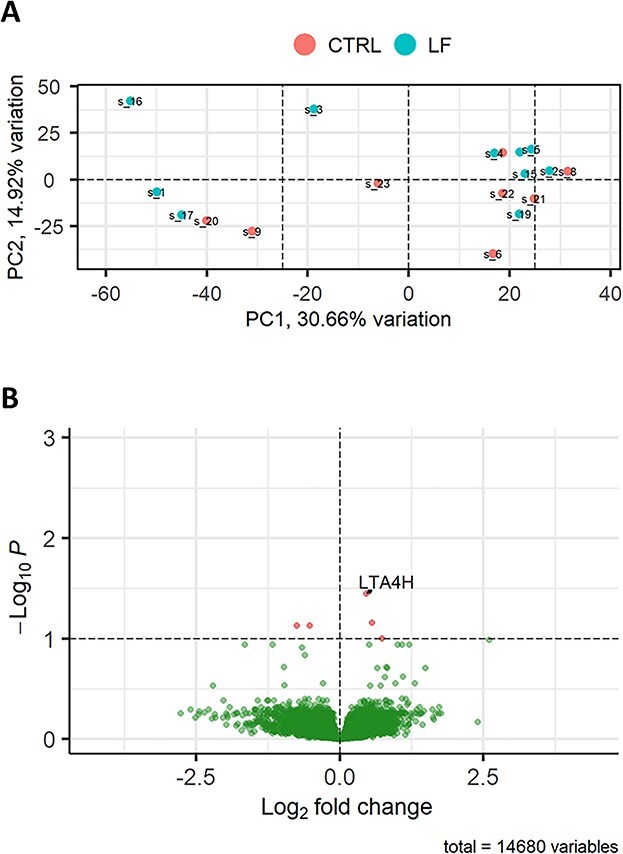
DEGs in the endometrium of heifers inseminated with frozen–thawed semen from LF (*n* = 10 heifers) bulls relative to the CTRL noninseminated heifers (*n* = 8 heifers). (A) PCA plot showing the distribution of RNA-seq samples, where colors indicate the two groups and numbers refer to specific heifer IDs (as detailed in Supplementary File 1). (B) Gene expression data are presented as a volcano plot using log values of the fold change and *P* value. Each data point represents a single gene, with those in red representing genes that survived the cut off thresholds of adjusted *P* < 0.05. The most significant DEGs are labelled, where gene names are available.

In contrast to the relatively high number of DEGs in the HF compared to the CTRL heifers, LF heifers had just 1 DEGs relative to the CTRL heifers. There was a reduced amount of segregation within groups as illustrated in the PCA plot (Figure 3). The low number of DEGs were evident in the volcano plot (Figure 3B). The fold change of the gene *LTA4H* was 1.37.

### Comparison 3: moderate but significant differential gene expression in the endometrium of heifers in response to semen from HF bulls relative to LF bulls

**Figure 4:**
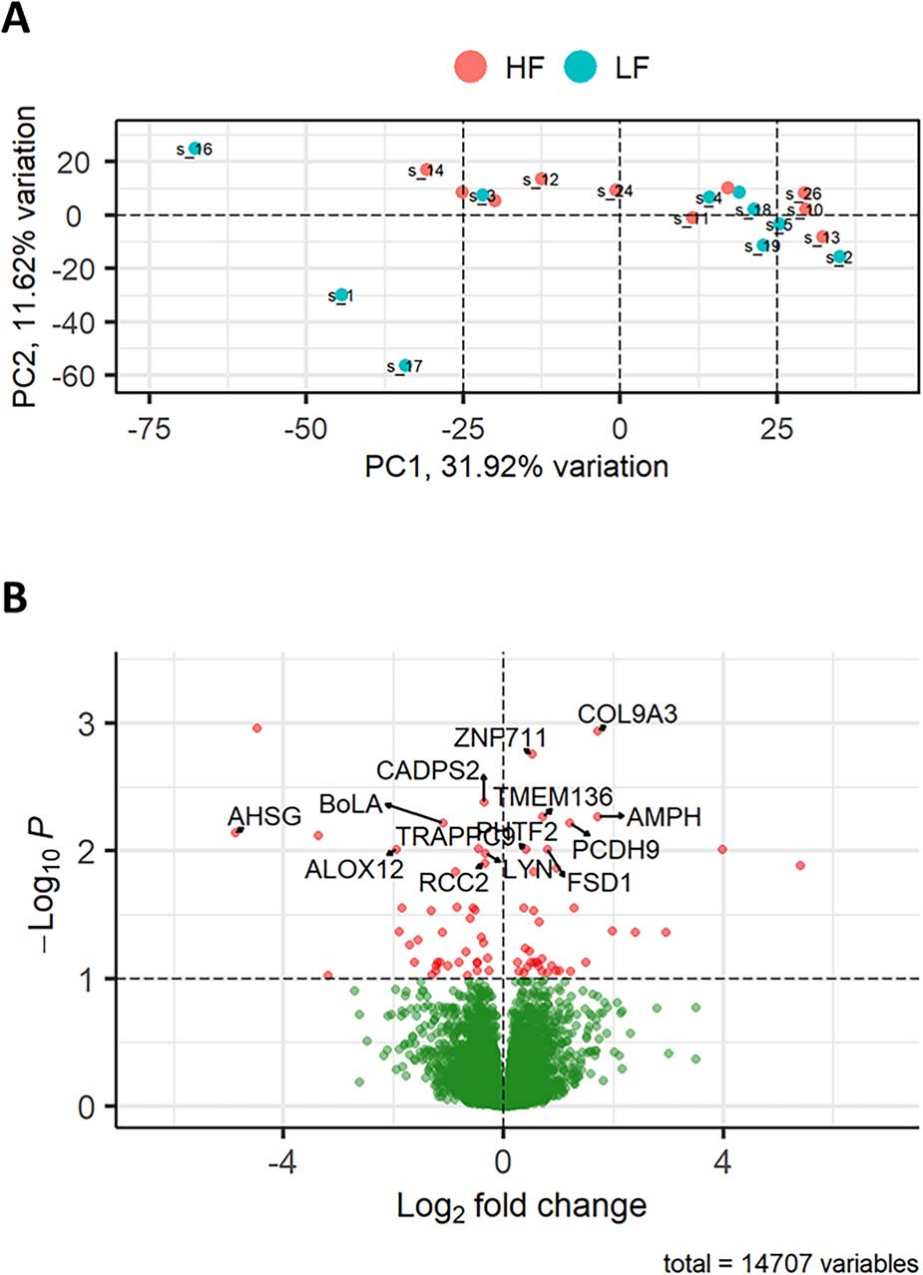
DEG) in the endometrium of heifers inseminated with frozen–thawed semen from HF bulls relative to LF bulls (*n* = 10 heifers per group). (A) PCA plot showing the distribution of RNA-seq samples, where colors indicate the two groups and numbers refer to specific heifer IDs (as detailed in Supplementary file 1. (B) Gene expression data are presented as a volcano plot using log values of the fold change and *P* value. Each data point represents a single gene, with those in red representing genes that survived the cut off thresholds of adjusted *P* < 0.05. The most significant differentially expressed genes are labelled, where gene names are available.

When the HF and LF treatments were directly compared, there were 40 DEGs in the endometrium of heifers inseminated with HF bulls relative to LF bulls. From the PCA plot, it is evident there is a mixed signal between the groups (Figure 4A) with an almost equal distribution of genes with increased expression in the HF treatment (20 genes) as the LF treatment (20 genes, Figure 4B). Fold changes that were increased in the HF relative to LF treatment varied from 1.29 to 42.18 (Supplementary file 2). The top 10 DEGs with increased expression in the HF treatment are shown in Table 4. While the highest gene expression belongs to the novel gene, it was followed by pro-inflammatory *IL1A.* The top 10 genes with lower expression in the HF treatment compared to the HF are displayed in Table 5. The fold change of the 20 genes decreased in HF relative to LF ranged from −29.50 to – 1.26.*AHSG* had the lowest expression in the HF treatment signifying a further role of the immune response [25].

**Table 4:** DEGs in the endometrium of heifers inseminated with frozen–thawed semen from HF bulls relative to LF bulls. Top 10 genes with positive absFC values indicating higher expression in the HF treatment.

**Gene symbol**	**Ensembl ID**	**Gene name**	**Gene functions**	**absFC**
	ENSBTAG00000047700	Novel gene		15.74
IL1A	ENSBTAG00000010349	Interleukin-1 Alpha	Cytokine activity; Immune response	5.57
AMPH	ENSBTAG00000031967	Amphiphysin	Regulation of exocytosis in synapses and certain endocrine cell types	3.27
COL9A3	ENSBTAG00000015581	Collagen type IX alpha 3 chain	Extracellular matrix structural constituent	3.27
PCDH9	ENSBTAG00000019340	Protocadherin 9	Calcium ion binding/cell adhesion	2.31
FSD1	ENSBTAG00000005999	Fibronectin type III and SPRY domain containing 1	Protein binding	1.73
TMEM136	ENSBTAG00000021342	TLC domain containing 5	Integral component of membrane	1.64
RUNX1T1	ENSBTAG00000017339	RUNX1 partner transcriptional Co-repressor 1	Facilitation of transcriptional repression	1.62
ZNF711	ENSBTAG00000002668	Zinc finger protein 711	Regulation if transcription	1.43
PHTF2	ENSBTAG00000005102	Putative homeodomain Transcription factor 2	Located in endoplasmic reticulum	1.32

**Table 5:** DEGs in the endometrium of heifers inseminated with frozen–thawed semen from HF relative to LF bulls. Top 10 genes with negative absFC values indicating lower expression in the HF treatment.

**Gene symbol**	**Ensembl ID**	**Gene name**	**Gene functions**	**absFC**
AHSG	ENSBTAG00000000522	Alpha 2-HS glycoprotein	Regulation of inflammatory response positive regulation of phagocytosis	−29.50
	ENSBTAG00000021077	Novel gene		−22.51
	ENSBTAG00000039289	Novel gene		−10.39
ALOX12	ENSBTAG00000021933	Arachidonate 12-lipoxygenase	Lipoxygenase activity	−3.85
BoLA	ENSBTAG00000005182	Major histocompatibility complex Class I. A	Antigen processing and presentation of endogenous peptide antigen via MHC class Ib	−2.14
WBSCR17	ENSBTAG00000008718	Polypeptide N-acetylgalactosaminyltransferase 17	Catalyzation of the initial reaction in O-linked oligosaccharide biosynthesis	−1.83
TRAPPC9	ENSBTAG00000013955	Trafficking protein particle complex 9	Positive regulation of NF-kappaB transcription factor activity	−1.37
CADPS2	ENSBTAG00000005110	Calcium-dependent secretion activator 2	Positive regulation of exocytosis synaptic vesicle exocytosis	−1.28
RCC2	ENSBTAG00000008579	Regulator of chromosome condensation 2	Facilitate normal progress through the cell cycle	−1.27
LYN	ENSBTAG00000020034	LYN Proto-Oncogene. Src family tyrosine kinase	The regulation of innate and adaptive immune responses. Hematopoiesis. Responses to growth factors and cytokines. Integrin signaling	−1.26

### Sperm induce a significant increase in neutrophil populations in the endometrium

**Figure 5:**
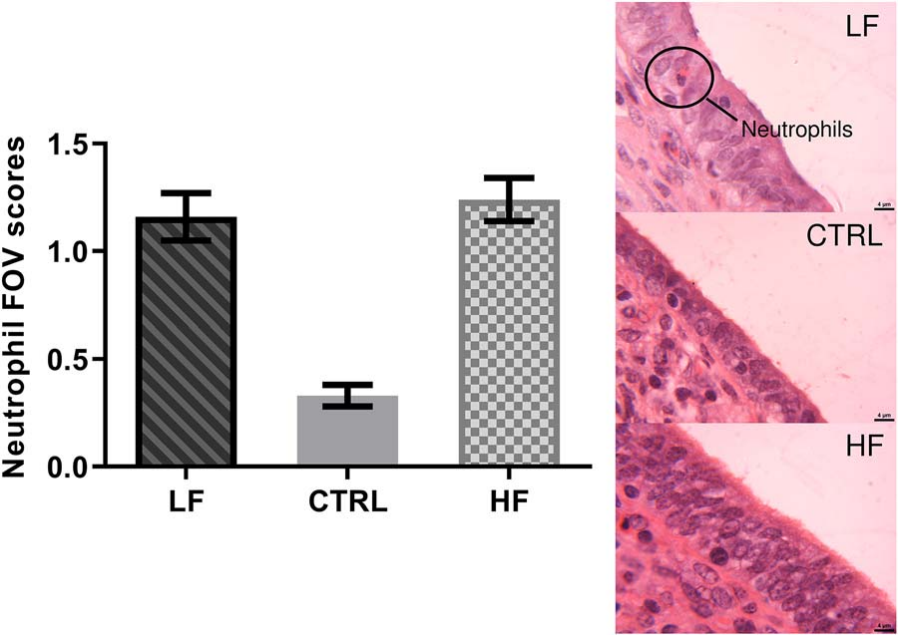
FOV scores of neutrophils in the endometrium of heifers inseminated with frozen–thawed semen from HF and LF bulls as well as a CTRL noninseminated treatment. Histological analysis of endometrium samples were performed on a subset of heifers (*n* = 3 per treatment). Tissue was stained with hematoxylin and eosin and assessed at 60x. Representative images from each treatment group are shown to the right. Scale bar = 4 μm. Data are presented as mean ± SEM.

There were more neutrophils in the epithelial layer of the endometrium in both the HF and LF treatments (average of 1.2 neutrophils per FOV) compared to the control group (average of 0.33 neutrophils per FOV; *P* < 0.05; Figure 5). However, there was no difference between the HF and LF treatments.

### *Interleukin-1 alpha* is localized mainly in the glandular epithelium

**Figure 6:**
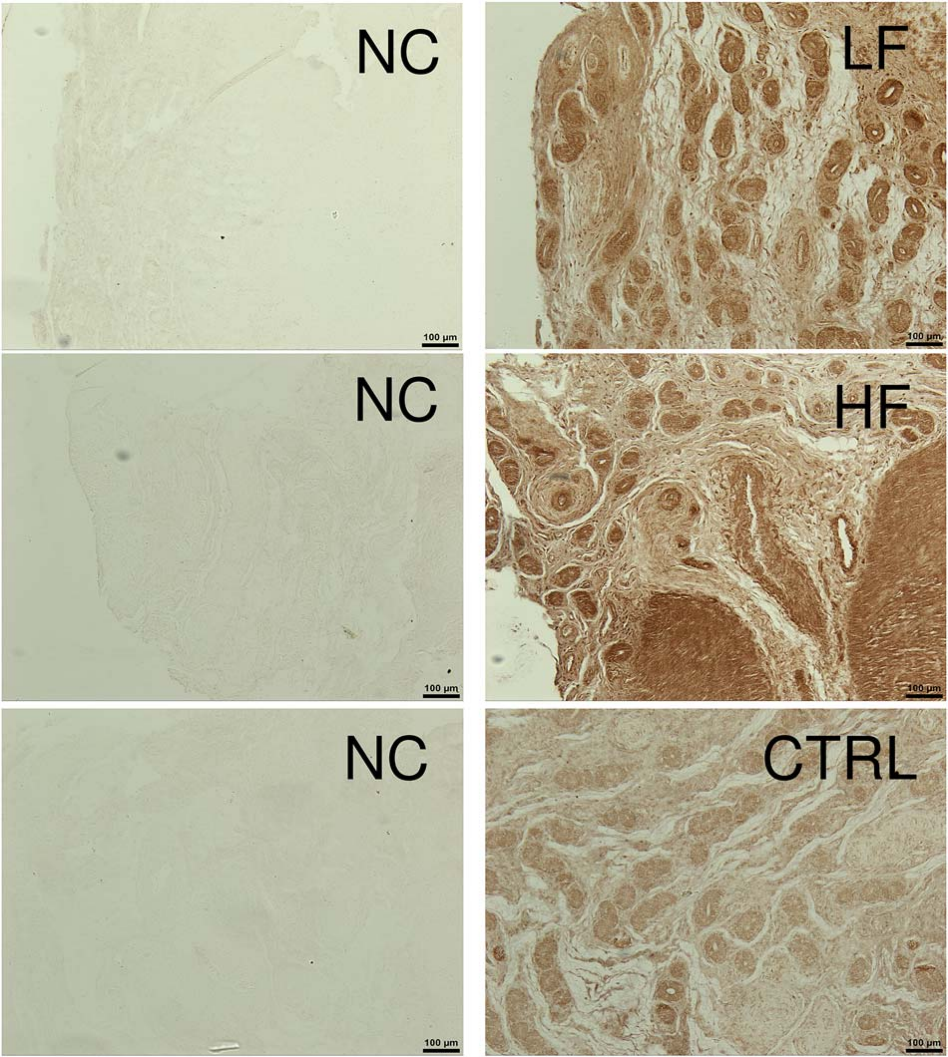
Representative pictures of immunohistochemical localization of IL1A in the endometrium of heifers inseminated with frozen–thawed semen from LF and HF bulls as well as a CTRL noninseminated treatment. Immunolocalization was performed on a subset of heifers (*n* = 3 per treatment). Tissue was imaged at 10x. Scale bar = 100 μm. IL1A is detectable by the presence of an insoluble brown precipitate in the tissue. Negative control (NC) was performed without primary antibody.

To verify localization of IL1A and assess if its expression differed between the endometrium of heifers inseminated with frozen–thawed semen from HF and LF bulls or not inseminated, immunohistochemistry was performed. While signal intensity of IL1A was strongest in the glandular epithelium, irrespective of fertility groups, its presence was also evident in the stromal tissue. The HF treatment appeared to have higher levels of IL1A expression compared to the LF treatment, while the CTRL had the lowest level of staining. Representative images are presented in Figure 6.

## Discussion

The main findings of this study were that not only are sperm immunogenic in the uterus but there was a differential endometrial transcriptomic response in response to sperm from bulls of high- and low-field fertility. When compared to the CTRL treatment, HF bulls had a more active transcriptomic response (376 DEGs) compared to the LF treatment (1 DEG). When directly compared, the HF relative to LF treatment had 40 DEGs, primarily immune genes, and genes associated with the inflammatory response.

The top genes with increased expression in the HF compared to CTRL treatment included *PTGDS, GABRA4*, and *SLC7A3. PTGDS*, also known as *L-PGDS*, has been shown to have an important role in the development of sperm and sperm maturation [26]. The top genes with increased expression in the CTRL compared to HF group included *ALOX12* and *RWDD4.* While *ALOX12* is known to regulate platelet aggregation and cell migration as well as inflammation, *RWDD4* does not have any specific known function [24]. Multiple genes from the solute carrier protein had significantly lower expression in the HF treatment. These are membrane integral proteins and are known facilitative and secondary active transporters [27]. GO analysis revealed a role in peptide binding, signaling receptor activator activity, and receptor ligand activity.

The top DEGs between the HF and LF groups directly, with increased expression in the HF group, include *IL1A, COL9A3, AMPH*, and *PDCH9. PCDH9* gene is from the photocadherin family with its main role to mediate cell-to-cell adhesion and in the recognition of the presence of calcium [28]. *COL9A3* is a collagen type IX alpha 3 chain, which is an extracellular matrix structural constituent [29]. *AMPH* is involved in actin polymerization, and thus play a role in cellular phagocytosis and endo/exocytosis [30]. This may be important for anti-inflammatory macrophages function as well as cytokine release in the uterus. Interestingly, *AMPH* is also involved in spermatids release from Sertoli cells in mice [31]. While a proportion of the DEGs are not linked directly with fertility, through stringent analysis of gene families, there is a clear prevalence of genes related to immunity. *IL1A* is a potent inflammatory marker and previous *in vitro* studies have shown sperm are capable of eliciting an inflammatory response in the uterus with an increase in pro-inflammatory molecules*CXCL8, TNFA*, and *IL1B* [12, 13]. In agreement with this, the signal intensity of IL1A from immunohistochemistry appeared to be stronger in the HF treatment when compared to LF treatment with minimal staining evident in the CTRL treatment. Its predominant presence in the glandular epithelium is likely due to the presence of sperm, which tend to induce stronger immune response in glandular epithelium. Moreover, IL1A is involved in the recruitment of PMNs [17]. The role of this sperm–neutrophil interaction is for the rapid removal of sperm from the endometrium in preparation for pregnancy [8, 32]. The increased inflammatory response from the HF bulls in this study could be associated with clearing and preparing the endometrium more effectively for pregnancy compared to LF bulls. Although, there was no difference in the number of PMNs recruited into the epithelial layer from heifers inseminated with HF or LF semen 12 h postinsemination. The most likely reason for this is that the recruitment of PMNs is a general response of the uterus to any invaders including sperm. Moreover, as PMNs can bind more than one sperm, we postulate that PMNs trapped more sperm from LF bulls as reported in our previous work [11], which could impede sperm transport to the site of fertilization. In a bovine *in vivo* study, a moderate influx of PMNs to the endometrium 4 h after insemination resulted in a significantly higher first service conception rate further emphasizing an immune focus on pregnancy rates [20]. Substantial populations of neutrophils have previously been reported to accumulate at implantation sites in mice, where they regulate implantation and tissue remodeling as well as linking the innate and adaptive immune responses [14, 33].

This study represents similarities to other bovine and murine *in vivo* studies where sperm plus SP but not SP alone altered the transcriptomic response *in vivo* [10, 14]. The top DEGs with decreased expression in HF compared to LF groups also include immune genes namely, *BOLA* (MHC class I), *AHSG, ALOX12*, and *TRAPPC9*. MHC molecules have been shown to be essential to the female’s tolerance to pregnancy establishment and although the genes expressed in this study are from 12 h postinsemination, the initial crosstalk between sperm and the female reproductive tract has been shown to be mediated by MHC class members [34, 35]. *AHSG* (known as Feutin) was also increased and has been shown to be an antagonist of the pro-inflammatory molecule *TNF* [25]. *ALOX12* is also related to the immune response although its exact role needs to be further elucidated. It has been proposed to be involved in human platelet aggregation as well chemotactic neutrophil migration [36, 37]. Furthermore, a study, which investigated the immune response of the cow endometrium to heat stress, reported an increased expression *ALOX12* in the group exposed to long-term heat stress [38]. *TRAPPC9* gene is known to play a key role in the NF-κB transcription factor, which involved a number of cellular processes such as innate and adaptive immunity, cellular proliferation, apoptosis, and development [39].

The extent of this immune response and its benefit on subsequent fertility is not exactly known; however, recent studies in mice show sperm signaling the adaptive immune response *in vivo* with the up-regulation of T-regulatory cells [14], which are important mediators of maternal immune tolerance and regulates embryo implantation and fetal survival [40]. Similarly, in this study, some genes in the wider dataset were significant and had strong read counts showing an increased expression of *IL11RA, IL4I1* as well as *IL1A* in the HF compared to CTRL group. While the role of *IL1A* was described earlier, the versatile role of *IL4I1* is also interesting. On one side, this interleukin inhibits T-cell activity, while on the other side, it positively regulates the function of B-cells [41]. Moreover, it has antimicrobial activity against both Gram-negative and Gram-positive bacteria, which may positively influence embryonic development [42] Indeed D’Amours et al. [43] found that IL4IA was more abundant in sperm from HF compared to LF bulls. In relation to *IL11RA*, it has been shown that null mutant female mice are infertile due to a defect in decidualization of the endometrium [15, 44].

In conclusion, this study has shown for the first time, a distinct divergent transcriptomic response in the endometrium of heifers inseminated with frozen–thawed semen from HF and LF bulls with a pivotal role of the inflammatory response. There was a clear effect of sperm in recruiting neutrophils to the endometrium with a more active transcriptomic response in the HF group, suggesting a more rapid clearing of the endometrium as well as preparing the endometrium for implantation and subsequent pregnancy. Considering the fact that semen induce an immune response not just in the endometrium but also in the oviduct, it would be interesting to elucidate the impact of sperm from bulls with divergent fertility on its immune response. In addition, to further our understanding of the effects of HF and LF sperm on pregnancy establishment, future studies should focus on priming of the endometrium with sperm and its effect on embryo development. This study not only furthers our understanding of the etiology of bull fertility but also of pregnancy establishment.

## Authors’ contributions

EMD identified the bulls, procured the bull semen, collected the uterine samples, and performed the extraction of total RNAs, the quality controls before sequencing, and drafted the manuscript. PC performed the bioinformatics and statistical analyses of mRNA data. GD and CR performed the histological assessments and immunohistochemistry. MS assisted in revising of the manuscript. KGM and SF conceived and obtained funding for the study and performed supervision of the work as well as critical revision of the manuscript. SF supplied the animals and synchronized them. All authors proof read and approved the final manuscript.

## Conflict of interest

Authors have declared no conflict of interest.

## Data availability

The datasets generated and/or analyzed during the current study are available in the NCBI Gene Expression Omnibus https://www.ncbi.nlm.nih.gov/geo/ under accession number GSE207735.

## Supplementary Material

Supplementary_1_Animal_info_ioad031

Supplementary_2_DEGs_ioad031

## References

[1] Fair S, Lonergan P. Review: understanding the causes of variation in reproductive wastage among bulls. Animal 2018; 12:s53–s62. 29779500 10.1017/S1751731118000964

[2] Utt MD . Prediction of bull fertility. Anim Reprod Sci 2016; 169:37–44. 26791329 10.1016/j.anireprosci.2015.12.011

[3] Bernecic NC, Donnellan E, O'Callaghan E, Kupisiewicz K, O'Meara C, Weldon K, Lonergan P, Kenny DA, Fair S. Comprehensive functional analysis reveals that acrosome integrity and viability are key variables distinguishing artificial insemination bulls of varying fertility. J Dairy Sci 2021; 104:11226–11241. 34253371 10.3168/jds.2021-20319

[4] Odhiambo JF, DeJarnette JM, Geary TW, Kennedy CE, Suarez SS, Sutovsky M, Sutovsky P. Increased conception rates in beef cattle inseminated with nanopurified bull Semen1. Biol Reprod 2014; 91:97. 25232015 10.1095/biolreprod.114.121897

[5] Sellem E, Broekhuijse ML, Chevrier L, Camugli S, Schmitt E, Schibler L, Koenen EP. Use of combinations of in vitro quality assessments to predict fertility of bovine semen. Theriogenology 2015; 84:1447–1454.e5. 26296523 10.1016/j.theriogenology.2015.07.035

[6] Holden SA, Fernandez-Fuertes B, Murphy C, Whelan H, O'Gorman A, Brennan L, Butler ST, Lonergan P, Fair S. Relationship between in vitro sperm functional assessments, seminal plasma composition, and field fertility after AI with either non-sorted or sex-sorted bull semen. Theriogenology 2017; 87:221–228. 27678515 10.1016/j.theriogenology.2016.08.024

[7] Miller DJ . Review: the epic journey of sperm through the female reproductive tract. Animal 2018; 12:s110–s120. 29551099 10.1017/S1751731118000526 PMC9556260

[8] Katila T . Post-mating inflammatory responses of the uterus. Reprod Domest Anim 2012; 47:31–41. 10.1111/j.1439-0531.2012.02120.x 22913558

[9] Christoffersen M, Troedsson M. Inflammation and fertility in the mare. Reprod Domest Anim 2017; 52:14–20. 28815848 10.1111/rda.13013

[10] Recuero S, Sánchez JM, Mateo-Otero Y, Bagés-Arnal S, McDonald M, Behura SK, Spencer TE, Kenny DA, Yeste M, Lonergan P, Fernandez-Fuertes B. Mating to intact, but not vasectomized, males elicits changes in the endometrial transcriptome: insights from the bovine model. Front Cell Dev Biol 2020; 8:547. 32766237 10.3389/fcell.2020.00547 PMC7381276

[11] Donnellan EM, O'Brien MB, Meade KG, Fair S. Comparison of the uterine inflammatory response to frozen-thawed sperm from high and low fertility bulls. Theriogenology 2021; 176:26–34. 34564014 10.1016/j.theriogenology.2021.09.012

[12] Elweza AE, Ezz MA, Acosta TJ, Talukder AK, Shimizu T, Hayakawa H, Shimada M, Imakawa K, Zaghloul AH, Miyamoto A. A proinflammatory response of bovine endometrial epithelial cells to active sperm in vitro. Mol Reprod Dev 2018; 85:215–226. 29337420 10.1002/mrd.22955

[13] Ezz MA, Marey MA, Elweza AE, Kawai T, Heppelmann M, Pfarrer C, Balboula AZ, Montaser A, Imakawa K, Zaabel SM, Shimada M, Miyamoto A. TLR2/4 signaling pathway mediates sperm-induced inflammation in bovine endometrial epithelial cells in vitro. PloS One 2019; 14:e0214516. 30995239 10.1371/journal.pone.0214516 PMC6469758

[14] Schjenken JE, Sharkey DJ, Green ES, Chan HY, Matias RA, Moldenhauer LM, Robertson SA. Sperm modulate uterine immune parameters relevant to embryo implantation and reproductive success in mice. Commun Biol 2021; 4:572. 33990675 10.1038/s42003-021-02038-9 PMC8121928

[15] Robb L, Li R, Hartley L, Nandurkar HH, Koentgen F, Begley CG. Infertility in female mice lacking the receptor for interleukin 11 is due to a defective uterine response to implantation. Nat Med 1998; 4:303–308. 9500603 10.1038/nm0398-303

[16] Minten MA, Bilby TR, Bruno RG, Allen CC, Madsen CA, Wang Z, Sawyer JE, Tibary A, Neibergs HL, Geary TW, Bauersachs S, Spencer TE. Effects of fertility on gene expression and function of the bovine endometrium. PloS One 2013; 8:e69444. 23940519 10.1371/journal.pone.0069444 PMC3734181

[17] Akthar I, Suarez S, Morillo VA, Sasaki M, Ezz MA, Takahashi KI, Shimada M, Marey MA, Miyamoto A. Sperm enter glands of preovulatory bovine endometrial explants and initiate inflammation. Reproduction 2019; 159:181–192. 10.1530/REP-19-0414 31794421

[18] Alghamdi AS, Lovaas BJ, Bird SL, Lamb GC, Rendahl AK, Taube PC, Foster DN. Species-specific interaction of seminal plasma on sperm-neutrophil binding. Anim Reprod Sci 2009; 114:331–344. 19081210 10.1016/j.anireprosci.2008.10.015

[19] Chastant S, Saint-Dizier M. Inflammation: friend or foe of bovine reproduction? Anim Reprod 2019; 16:539–547. 32435296 10.21451/1984-3143-AR2019-0057 PMC7234060

[20] Kaufmann TB, Drillich M, Tenhagen BA, Forderung D, Heuwieser W. Prevalence of bovine subclinical endometritis 4h after insemination and its effects on first service conception rate. Theriogenology 2009; 71:385–391. 18801562 10.1016/j.theriogenology.2008.08.005

[21] Berry DP, Evans RD, Mc PS. Evaluation of bull fertility in dairy and beef cattle using cow field data. Theriogenology 2011; 75:172–181. 20875673 10.1016/j.theriogenology.2010.08.002

[22] Yu G, Wang LG, Han Y, He QY. clusterProfiler: an R package for comparing biological themes among gene clusters. OMICS 2012; 16:284–287. 22455463 10.1089/omi.2011.0118 PMC3339379

[23] Burkhard P, Stetefeld J, Strelkov SV. Coiled coils: a highly versatile protein folding motif. Trends Cell Biol 2001; 11:82–88. 11166216 10.1016/s0962-8924(00)01898-5

[24] Zheng Z, Li Y, Jin G, Huang T, Zou M, Duan S. The biological role of arachidonic acid 12-lipoxygenase (ALOX12) in various human diseases. Biomed Pharmacother 2020; 129:110354. 32540644 10.1016/j.biopha.2020.110354

[25] Ombrellino M, Wang H, Yang H, Zhang M, Vishnubhakat J, Frazier A, Scher LA, Friedman SG, Tracey KJ. Fetuin, a negative acute phase protein, attenuates TNF synthesis and the innate inflammatory response to carrageenan. Shock 2001; 15:181–185. 11236900 10.1097/00024382-200115030-00004

[26] Rossitto M, Ujjan S, Poulat F, Boizet-Bonhoure B. Multiple roles of the prostaglandin D2 signaling pathway in reproduction. Reproduction 2015; 149:R49–R58. 25269616 10.1530/REP-14-0381

[27] Cesar-Razquin A, Snijder B, Frappier-Brinton T, Isserlin R, Gyimesi G, Bai X, Reithmeier RA, Hepworth D, Hediger MA, Edwards AM, Superti-Furga G. A call for systematic research on solute carriers. Cell 2015; 162:478–487. 26232220 10.1016/j.cell.2015.07.022

[28] Wang C, Chen Q, Li S, Zhao Z, Gao H, Wang X, Li B, Zhang W, Yuan Y, Ming L, He H, Tao B, et al. Dual inhibition of PCDH9 expression by miR-215-5p up-regulation in gliomas. Oncotarget 2017; 8:10287–10297. 28055966 10.18632/oncotarget.14396 PMC5354659

[29] Huang D, Deng X, Ma K, Wu F, Shi D, Liang H, Chen S, Shao Z. Association of COL9A3 trp3 polymorphism with intervertebral disk degeneration: a meta-analysis. BMC Musculoskelet Disord 2018; 19:381. 30342505 10.1186/s12891-018-2297-y PMC6195691

[30] Yamada H, Ohashi E, Abe T, Kusumi N, Li SA, Yoshida Y, Watanabe M, Tomizawa K, Kashiwakura Y, Kumon H, Matsui H, Takei K. Amphiphysin 1 is important for actin polymerization during phagocytosis. Mol Biol Cell 2007; 18:4669–4680. 17855509 10.1091/mbc.E07-04-0296 PMC2043535

[31] Kusumi N, Watanabe M, Yamada H, Li SA, Kashiwakura Y, Matsukawa T, Nagai A, Nasu Y, Kumon H, Takei K. Implication of amphiphysin 1 and dynamin 2 in tubulobulbar complex formation and spermatid release. Cell Struct Funct 2007; 32:101–113. 17785912 10.1247/csf.07024

[32] Hansen PJ . The immunology of early pregnancy in farm animals. Reprod Domest Anim 2011; 46:18–30. 10.1111/j.1439-0531.2011.01850.x 21854458

[33] Hall CHT, Campbell EL, Colgan SP. Neutrophils as components of mucosal homeostasis. Cell Mol Gastroenterol Hepatol 2017; 4:329–337. 28884136 10.1016/j.jcmgh.2017.07.001 PMC5581871

[34] Rapacz-Leonard A, Dąbrowska M, Janowski T. Major histocompatibility complex I mediates immunological tolerance of the trophoblast during pregnancy and may mediate rejection during parturition. Mediators Inflamm 2014; 2014:579279. 24812442 10.1155/2014/579279 PMC4000645

[35] Melo TP, de Camargo GMF, de Albuquerque LG, Carvalheiro R. Genome-wide association study provides strong evidence of genes affecting the reproductive performance of Nellore beef cows. PloS One 2017; 12:e0178551. 28562680 10.1371/journal.pone.0178551 PMC5451131

[36] Loynes CA, Lee JA, Robertson AL, Steel MJ, Ellett F, Feng Y, Levy BD, Whyte MKB, Renshaw SA. PGE2 production at sites of tissue injury promotes an anti-inflammatory neutrophil phenotype and determines the outcome of inflammation resolution in vivo. Sci Adv 2018; 4:eaar8320. 30191175 10.1126/sciadv.aar8320 PMC6124908

[37] Chen XS, Funk CD. Structure-function properties of human platelet 12-lipoxygenase: chimeric enzyme and in vitro mutagenesis studies. FASEB J 1993; 7:694–701. 8500694 10.1096/fasebj.7.8.8500694

[38] Chotimanukul S, Suwimonteerabutr J, Techakumphu M, Swangchan-Uthai T. In vitro effects of short-term and long-term heat exposures on the immune response and prostaglandin biosynthesis in bovine endometrial cells. Animals (Basel) 2022; 12. 10.3390/ani12182359. PMC9495028 36139219

[39] Zhang Q, Lenardo MJ, Baltimore D. 30 years of NF-κB: a blossoming of relevance to human pathobiology. Cell 2017; 168:37–57. 28086098 10.1016/j.cell.2016.12.012 PMC5268070

[40] Samstein RM, Josefowicz SZ, Arvey A, Treuting PM, Rudensky AY. Extrathymic generation of regulatory T cells in placental mammals mitigates maternal-fetal conflict. Cell 2012; 150:29–38. 22770213 10.1016/j.cell.2012.05.031 PMC3422629

[41] Yue Y, Huang W, Liang J, Guo J, Ji J, Yao Y, Zheng M, Cai Z, Lu L, Wang J. IL4I1 is a novel regulator of M2 macrophage polarization that can inhibit T cell activation via L-tryptophan and arginine depletion and IL-10 production. PloS One 2015; 10:e0142979. 26599209 10.1371/journal.pone.0142979 PMC4658051

[42] Puiffe ML, Lachaise I, Molinier-Frenkel V, Castellano F. Antibacterial properties of the mammalian L-amino acid oxidase IL4I1. PloS One 2013; 8:e54589. 23355881 10.1371/journal.pone.0054589 PMC3552961

[43] D'Amours O, Calvo E, Bourassa S, Vincent P, Blondin P, Sullivan R. Proteomic markers of low and high fertility bovine spermatozoa separated by Percoll gradient. Mol Reprod Dev 2019; 86:999–1012. 31134708 10.1002/mrd.23174

[44] Dimitriadis E, Robb L, Liu YX, Enders AC, Martin H, Stoikos C, Wallace E, Salamonsen LA. IL-11 and IL-11Ralpha immunolocalisation at primate implantation sites supports a role for IL-11 in placentation and fetal development. Reprod Biol Endocrinol 2003; 1:34. 12740032 10.1186/1477-7827-1-34 PMC155642

